# Immediate Sequential Bilateral Cataract Surgery: A Systematic Review and Meta-Analysis

**DOI:** 10.1155/2015/912481

**Published:** 2015-08-17

**Authors:** Line Kessel, Jens Andresen, Ditte Erngaard, Per Flesner, Britta Tendal, Jesper Hjortdal

**Affiliations:** ^1^Department of Ophthalmology, Rigshospitalet-Glostrup, 2600 Glostrup, Denmark; ^2^Danish Health and Medicines Authority, 2300 Copenhagen S, Denmark; ^3^Skanderborg Eye Clinic, 8660 Skanderborg, Denmark; ^4^Department of Ophthalmology, Næstved Hospital, 4700 Næstved, Denmark; ^5^Odense Eye Clinic, 5000 Odense C, Denmark; ^6^Department of Ophthalmology, Aarhus University Hospital NBG, 8000 Aarhus, Denmark

## Abstract

The aim of the present systematic review was to examine the benefits and harms associated with immediate sequential bilateral cataract surgery (ISBCS) with specific emphasis on the rate of complications, postoperative anisometropia, and subjective visual function in order to formulate evidence-based national Danish guidelines for cataract surgery. A systematic literature review in PubMed, Embase, and Cochrane central databases identified three randomized controlled trials that compared outcome in patients randomized to ISBCS or bilateral cataract surgery on two different dates. Meta-analyses were performed using the Cochrane Review Manager software. The quality of the evidence was assessed using the GRADE method (Grading of Recommendation, Assessment, Development, and Evaluation). We did not find any difference in the risk of complications or visual outcome in patients randomized to ISBCS or surgery on two different dates. The quality of evidence was rated as low to very low. None of the studies reported the prevalence of postoperative anisometropia. In conclusion, we cannot provide evidence-based recommendations on the use of ISBCS due to the lack of high quality evidence. Therefore, the decision to perform ISBCS should be taken after careful discussion between the surgeon and the patient.

## 1. Introduction

Cataract surgery is one of the most commonly performed elective surgical procedures in most Westernized countries. In the US Medicare system, cataract is the second most expensive procedure after intravitreal injections of anti-VEGF [1]. With the growing number of older citizens, the need for eye care is expected to rise [2]. The need for cataract surgery alone is expected to double within the next 20 years [3]. We need to prioritize resources to be able to provide service to those most at need.

Immediate sequential bilateral cataract surgery (ISBCS), that is, surgery performed on both eyes on the same day but as separate procedures, has caused some controversy. Those in favor of the procedure argue that the postoperative visual rehabilitation period is faster and that fewer visits to the clinic or hospital are needed, which saves money and time for both health professionals and patients [4–6]. Those, who object to the procedure, argue that the risk of bilateral sight-threatening complications and the risk of postoperative refractive surprises outweigh any potential benefits that the procedure may have [7, 8]. If the two surgeries are performed independently with strict hygienic precautions (e.g., rescrubbing of lids, redraping, regowning, and separate batches of surgical devices), the risk of bilateral endophthalmitis is small [9]. Reimbursement practices may also affect the likelihood of a surgeon considering ISBCS or bilateral cataract surgery on two separate days [10].

A study from Sweden found that delayed sequential bilateral cataract surgery, that is, surgery on both eyes but on separate dates, was 14% more expensive than ISBCS [11]. A Finnish study considering both the direct costs related to the surgery and transportation and time costs for the patient found that delayed sequential bilateral surgery was 849 Euros more expensive than ISBCS [12].

Thus, ISBCS may have its economic advantages but it may be at an expense of bilateral severe complications to a few patients. How do we balance the benefits and risks? With the increasing need for cataract surgery and a shortage of health care resources as well as the lessons learned from corneal refractive surgery where bilateral procedures are usually performed successfully on the same day, we felt that it was relevant to reconsider whether ISBCS can be performed safely. The present study is a systematic review of the existing literature aimed at evaluating the safety aspects, risk, and benefits associated with ISBCS. The work was undertaken after an initiation by the Danish National Health and Medicines Authority to formulate evidence-based national guidelines on surgery for age-related cataract.

## 2. Methods

The aim of the present systematic review was to examine the benefits and harms associated with immediate sequential bilateral cataract surgery (ISBCS) with specific emphasis on the rate of complications, postoperative anisometropia, and subjective visual function in order to formulate evidence-based national Danish guidelines for cataract surgery. The review and resulting meta-analysis were performed based on the principles described in the Grading of Recommendation, Assessment, Development, and Evaluation (GRADE) approach [13]. The first step in the working process was to define the important questions and decide how to evaluate those questions using the PICO approach [14]. In short, PICO stands for patient, intervention, comparison, and outcome. For this specific review and meta-analysis, we chose to examine the risks and advantages of ISBCS for patients with bilateral age-related cataract undergoing phacoemulsification (P). We extracted data from references where the patients were randomized to ISBCS (I) or surgery on separate days (C). As outcome measures (O), we decided on the number of any adverse events, serious adverse events (specifically the number of sight-threatening complications), and postoperative anisometropia (>2 diopters difference in spherical equivalent) as well as the patient's subjective satisfaction with the procedure.

A systematic literature search was conducted in September 2014 in the Embase and PubMed.gov databases and the Cochrane Central database using the search term: ((((immediate sequential) OR bilateral surgery) OR same-day)) AND (((cataract) OR cataract extraction) OR cataract surgery). The search was limited to references published within the last 10 years in the English or Scandinavian languages. The search yielded 801 hits. References were screened by title and abstract for eligibility. If there was any doubt as to the eligibility of the reference, the reference was obtained and read in full.

Study characteristics and outcome data were assessed and extracted independently by two authors (Line Kessel and Jesper Hjortdal). Cases of disagreement were solved by discussion and consensus. Risk of bias of the included studies was evaluated using the Cochrane Risk of Bias tool [15] in the Review Manager 5 Software [16]. In short, the Cochrane Risk of Bias tool assesses risk of bias associated with the selection of patients (randomization or patient allocation and concealment of allocation), study performance (blinding of patients and personnel), detection of outcomes (blinding of outcome assessment), attrition of data (such as missing patients or drop-outs), reporting of study findings (selective outcome reporting), or other types of bias related to the study design that could affect the internal validity.

The quality of the evidence for each outcome was evaluated across the included studies and evidence profiles were prepared using the GRADE profiler software [17]. The available evidence was assessed for study limitations (risk of bias, e.g., lack of allocation concealment or lack of blinding of patients or outcome assessors, incomplete accounting of patients and outcome, selective outcome reporting, or other limitations) [18], inconsistency (different results between studies) [19], indirectness (which was the study population and intervention comparable to the patient population and intervention, i.e., relevant to the readers of meta-analysis and use of surrogate measures) [20], imprecision (large confidence intervals (CI) or the lack of statistical strength by included studies to answer the posed question) [21], and risk of publication bias (small number of studies or small number of included patients and lack of reporting of negative findings) [22].

Continuous data were analyzed according to differences in mean treatment effects and their standard deviations. Dichotomous outcome data were analyzed by calculating risk ratios (RR). The Review Manager 5 Software [16] was used for estimation of overall effects. We used random-effects models to calculate pooled estimates of effects.

According to Danish law, no ethical committee or institutional board approval was required for this study.

## 3. Results

Three randomized controlled clinical trials (RCTs) examining the safety and efficacy of ISBCS versus cataract surgery performed on two different days were identified by a systematic review of the literature [23–25]. Furthermore, 27 observational studies were identified (including retrospective and prospective cohort studies). The RCTs were included in the meta-analysis and all other study types were excluded. The characteristics of included studies are presented in Table 1. Risk of bias assessment for included studies is provided in Table 2. A list of excluded studies with reasons for exclusion is provided in Table 3. All three included RCTs only included patients without competing eye diseases and with a limited range of axial lengths. All surgeries were performed by experienced surgeons. In the following, we analyze the safety of ISBCS compared to surgery performed on two different days with special emphasis on intra- and postoperative complications, postoperative anisometropia, and patient satisfaction.

### 3.1. Risk of Any Intra- or Postoperative Complication

**Figure 1:**
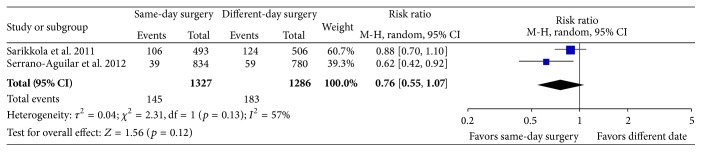
Forest plot of any intra- or postoperative complication (including sensation of dry eyes). M-H: Mantel-Haenszel. CI: confidence interval.

Two of the included RCTs provided information on the number of complications (peri- and postoperative) in the two groups randomized to ISBCS or surgery on different days [24, 25]. The third study provided information on the total rate of complications for the two groups combined but not for each group separately (6/96 = a complication rate of 6.3%, including high intraocular pressure < 30 mmHg on the first postoperative day in 2 eyes and one with a corneal edema; at 2 months postoperatively 1 eye had iritis and at 4 months one eye had a vitreous detachment and 2 eyes (1 patient) had beginnings of posterior capsule opacification) [23]. The reported prevalence of postoperative complications was markedly different in the remaining two studies [24, 25], appearing to reflect different opinions in what was considered a postoperative complication; for example, only one of the studies included sutures in wound, first day postoperative pressure rise > 30 mmHg, or signs of posterior capsule fibrosis in the list of complications. Looking at any complication (intra- or postoperatively within the first month) the two studies [24, 25] reported a complication rate of 23% and 6%, respectively. The reported complications were capsule tears (*n* = 17), vitreous loss (*n* = 5), iridectomy or sphincterotomy (*n* = 7), sutures in wound (*n* = 34), intraocular pressure > 30 mmHg on the first postoperative day (*n* = 67), wound leak (*n* = 2), IOL decentration or deplacement (*n* = 6), and corneal edema (*n* = 31) and after one month IOL decentration (*n* = 2), corneal edema (*n* = 13), anterior chamber flare (*n* = 7), capsular fibrosis (*n* = 36), and macular edema (*n* = 3) in one study [25] and iris prolapse (*n* = 2), posterior capsule tear (*n* = 1), corneal edema on first postoperative day (*n* = 13), capsule opacification (*n* = 1) and foreign body sensation (*n* = 1), and dry eyes (*n* = 80) in the other study [24]. There was a tendency towards lower number of complications in the groups randomized to ISBCS (RR (95% CI) 0.76 (0.55, 1.07), *p* = 0.12, see Figure 1). Due to the large inconsistency in number of reported complications and the fact that the outcome assessors in the included RCTs were not blinded to patient randomization, the quality of the evidence was rated as very low; see Table 4 for a summary of the evidence and quality of the evidence assessment.

Most surgeons performing ISBCS would recommend deferring surgery on the second eye in case of intraoperative complications. In two of the included RCTs, none of the patients required deferral of second eye surgery because of intraoperative complications [24, 25]. In the last study, three patients were excluded because of intraoperative complications [23]. Thus, three ISBCS patients out of 1377 (0.2%) had to have their second eye surgery deferred because of intraoperative complications.

### 3.2. Rate of Serious Complications

**Figure 2:**
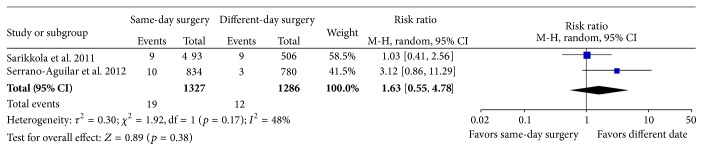
Forest plot of number of serious postoperative complications (corneal edema, macular edema, wound leak, or iris prolapse) detected within the first month. M-H: Mantel-Haenszel. CI: confidence interval.

None of the studies included enough patients to be able to detect rare but serious side effects and sight-threatening complications. Instead, we evaluated serious complications as the complications that could potentially be of threat to visual outcome, for example, corneal edema, macular edema, wound leakage, or iris prolapse. In total, the number of serious complications found within the three included RCTs was 26 with corneal edema, three with macular edema, two with wound leakage, and 0 with iris prolapse. The rate of serious complications detected within the first postoperative month was 0.8% [24] and 1.8% [25], respectively. There was no significant difference in the rate of serious postoperative complications between patients randomized to ISBCS or surgery on different days (*p* = 0.38); see Figure 2. One study reevaluated the hospital files one year after termination of the study and did not find any cases of retinal detachment within the first year postoperatively [25]. Due to the fact that the outcome assessors were not blinded to the patients' randomization status and that the studies were not large enough to assess serious complications, the quality of the evidence was rated as low; see Table 4.

### 3.3. Postoperative Anisometropia (>2 Diopters)

None of the included studies reported the number of patients who ended up with postoperative anisometropia of 2 diopters or greater, nor did they report lower grades of anisometropia. One study reported the mean difference between eyes in spherical equivalent after bilateral surgery and found that it was around 0.5 D but the range was not reported [23]. A second study found that the postoperative refraction was within 1 day of the target at 1 month after surgery in around 90% of patients in both the ISBCS and the different date groups [25]. Thus, none of the included studies could provide evidence as to the prevalence of postoperative anisometropia in patients undergoing ISBCS.

### 3.4. Subjective Satisfaction with Visual Function

**Figure 3:**
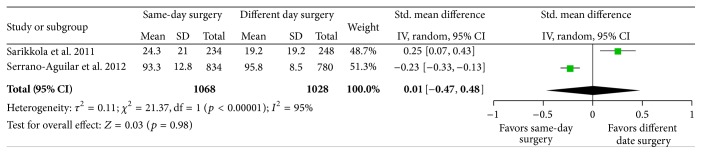
Subjective visual function assessed using the VF-7 (Sarikkola) or VF-14 (Serrano-Aguilar) questionnaire 1 month after bilateral cataract surgery in patients randomized to immediate sequential bilateral cataract surgery (same-day surgery) or different date bilateral cataract surgery. CI: confidence interval. IV: inverse variance. SD: standard deviation.

All three included RCTs reported the subjective satisfaction with visual function postoperatively but one study did not report the standard deviation; therefore, we could not include it in the meta-analysis [23]. The remaining two studies [24, 25] evaluated visual function on two different scales (VF-7 and VF-14) and hence we used the standardized means method in order to include both the studies in the same meta-analysis. In the group randomized to bilateral surgery on two different days, subjective visual function was lower in the period between first and second eye surgeries. This effect disappeared when the second eye was operated on and 1-2 months after bilateral surgery there was no difference in subjective visual function between the groups randomized to ISBCS or surgery on two different days; see Figure 3. Since none of the studies was blinded, the quality of the evidence was rated as moderate; see Table 3.

## 4. Discussion

Immediate sequential bilateral cataract surgery is a matter of controversy with strong arguments against and in favor of the procedure. In some countries, such as the Spanish Canary Islands, Sweden, and Finland, the procedure is widely accepted and a large proportion of cataract patients are operated on both eyes on the same date. In other countries, ISBCS is rarely performed even in patients undergoing general anaesthesia. The present study was conducted to provide evidence-based recommendations on the risks associated with ISBCS. Since we wanted to provide evidence of the highest possible quality [18, 26], we chose only to include randomized trials comparing ISBCS to bilateral cataract surgery performed on two different days. A review based on nonrandomized trials reporting the outcome after ISBCS was published by others [27]. However, even though we only included randomized trials, the level of evidence was low to moderate. Thus, there is no strong scientific background to advice against or in favor of ISBCS.

After a systematic literature search, we identified three RCTs including a total of 1900 patients. Even though we restricted our analyses to randomized studies, the quality of evidence for each outcome across the trials ranged from low to moderate. One reason for rating down the quality of the evidence was that outcome assessment was not blinded in any of the studies. It was an inherent part of the study design that neither surgeons nor patients could be blinded as to whether both eyes were operated on the same day or on separate dates. The assessment of outcome at follow-up could, however, easily have been blinded but the studies provided no information as to whether this was the case or not. Future studies could be designed with a follow-up of 3 to 6 months and the person assessing the outcome at final follow-up could be blinded to when each eye was operated on. In addition, statistical analyses can be performed with masking of data, so that the person performing the statistical analyses is blinded to which intervention each group of patients was randomized to. Furthermore, the quality of evidence was rated down for inconsistency due to the large differences in reported number of complications. This probably reflects differences in opinion regarding what is considered a complication but nevertheless there is reason for concern when raised intraocular pressure on the first postoperative day is found in 7% of patients in one study [25] but in no patients in a second study [24]. Others have found pressure rise rates on the first postoperative day around 8% [28]. Finally, the quality of the evidence was rated down because the three RCTs in combination had included too few patients to evaluate serious postoperative complications with any certainty; for example, the rate of endophthalmitis is between 0.175% (in the ESCRS study [29]) and 0.029% (in Sweden [30]).

One of the strongest arguments against ISBCS is the risk of bilateral endophthalmitis. To follow this argument, the second eye should not be operated on before the first eye is safely beyond the risk of endophthalmitis. In the ESCRS study, four of the 29 cases (14%) of endophthalmitis were diagnosed later than 2 weeks after surgery with two cases presenting at day 36 (negative culture) and day 132 (*Propionibacterium acnes*) [31]. This suggests that a significant amount of time should lapse between surgeries to assure that no patient has bilateral endophthalmitis. Bilateral endophthalmitis [32, 33] and bilateral early corneal decompensation requiring corneal transplantation [34] have been both described after ISBCS but the overall rate of endophthalmitis is not expected to be higher after ISBCS compared to surgery on two different days [35]. In one of the cases of bilateral endophthalmitis [33], surgical procedures were not optimized for ISBCS with reuse of irrigating fluids and flash sterilization [36] and in the other study instruments were autoclaved but the quality of the sterilization procedure was not checked [32].

The risk of retinal detachment increases markedly after cataract surgery [37, 38] but the time lapse between surgery and retinal detachment means that if it occurs, the retinal detachment will not be diagnosed in due time before second eye surgery even if bilateral surgery is performed on separate days [27]. The evidence presented in the present systematic review does not allow for any conclusions to be drawn as to whether the risk of complications is higher or lower after ISBCS than different date bilateral cataract surgery due to the low quality of evidence.

Part of the argument against ISBCS is based on the risk of refractive surprises. If surgeries are performed on two different days, the refractive outcome of the first eye can be used to guide the refractive plan for the second eye. A study, where the biometry was based on an ultrasound application method and where the difference in axial length between eyes was very large, found that the refractive outcome of the first eye was not of value in adjusting the refractive plan of the second eye [39]. Using newer optically based methods of axial length determination, the refractive prediction of the second eye was, however, improved when information from the first eye was used [40, 41]. None of the included RCTs provided information as to the postoperative anisometropia after bilateral surgery. A retrospective study evaluating the prevalence of postoperative anisometropia in patients undergoing ISBCS found that postoperative anisometropia was >2 diopters in 1.2% of patients [42]. To minimize the risk of postoperative anisometropia and unwanted refractive surprises, two of the included RCTs excluded patients with axial lengths outside the normal range [23, 25].

Some careful consideration is required in patients with high refractive errors; longer time between surgeries means longer periods of poor visual function due to postoperative anisometropia. Most centers performing corneal refractive surgery offer surgery on both eyes on the same day to avoid postoperative anisometropia. From a surgeon's point of view, it seems advisable to limit ISBCS to the group of patients with low expected risk of peri- or postoperative complications and this may exclude some patients with extreme refractive errors. However, from a patient's point of view, the time lapse between surgeries should be as short as possible especially if the patient has high refractive errors because of the large anisometropia when one eye has had surgery and the other has not.

None of the included studies evaluated the subjective satisfaction of undergoing cataract surgery as an immediate or delayed sequential bilateral procedure. From a patient perspective, ISBCS may be an advantage due to the faster optical rehabilitation. A retrospective study found that a significant majority of patients (90%) would recommend or recommend with pleasure ISBCS to their relatives or friends whereas only 2% would not recommend the procedure [43].

All three RCTs evaluated the subjective visual function postoperatively. None of the studies found a significant difference in subjective visual function after bilateral surgery between patients randomized to ISBCS or different date bilateral cataract surgery [23–25]. In patients who were randomized to different date surgery, there was a poorer self-assessed visual function in the time period between first and second eye surgeries. The difference disappeared after second eye surgery. Based on our results, we cannot say that one method provides better subjective visual outcome than the other method or that one method should be recommended over the other based on subjective visual function.

The previous Danish guideline for cataract surgery published by the Danish Ophthalmological Society in 2001 stated that ISBCS should not be performed because of a lack of evidence on the safety aspects of the procedure [44]. The American Academy of Ophthalmology does not advice for or against ISBCS [45]. The Royal Society of Ophthalmologists is generally cautious about performing ISBCS but advise that it may be performed in patients with a need for general anaesthesia and in whom repeated general anaesthesia is contraindicated for medical reasons [46].

In summary, we found that there was scientific evidence of very low to moderate quality regarding the risks and benefits of ISBCS. We did not find reason to suspect that complications were more or less frequent after ISBCS but we cannot rule out that they were. The effect on postoperative anisometropia could not be evaluated by the included randomized trials but a retrospective study indicated that this would not be a major problem. Self-assessed visual function was the same after bilateral surgery no matter if patients were operated bilaterally on the same day or 2 months apart but poorer in the time interval between the surgeries for patients operated on different days. Performing cataract surgery with a time interval between the two eyes allows for detection of some of the sight-threatening complications such as early endophthalmitis, but cystoid macular edema, retinal detachment, and late corneal decompensation usually present with a greater time lag than what most surgeons use between the two surgical procedures. If immediate sequential bilateral surgery is performed, it seems pertinent that the two surgeries are performed as two separate procedures including regowning of the surgeon and assistant, redraping and cleaning of the eye region, refitting the surgical equipment, and possibly also using two separate batches of viscoelastica and IOLs. Intracameral antibiotics significantly lower the risk of endophthalmitis [47] and it seems mandatory that intracameral antibiotics should be used if ISBCS is performed. In Sweden, where around 6% of cataract surgeries are performed as same-day procedure, two intracameral antibiotics (cefuroxime and ampicillin) are used during ISBCS in order to minimize the risk of endophthalmitis [30] and since no cases of bilateral endophthalmitis after same-day surgery have been reported in Sweden (Mats Lundström, personal communication), this seems to be a good advice.

## 5. Conclusions and Recommendations

Immediate sequential bilateral cataract surgery may offer some advantages in terms of saving of health resources and faster optical rehabilitation but it is at the risk of simultaneous, bilateral complications. The level of evidence concerning ISBCS is low and hence it is not possible to formulate an evidence-based recommendation. Immediate sequential bilateral cataract surgery may be a good option for patients undergoing surgery in general anaesthesia and in whom repeated general anaesthesia is associated with increased health risks. Any general or ocular condition that might increase the risk for any peri- or postoperative complication conflicts with the use of ISBCS. Patients should be informed of and have consented to the risks associated with ISBCS and it should only be performed by experienced surgeons taking meticulous care to adhere to strict hygienic standards with the two procedures being performed as independent procedures including redraping and regowning and with the use of separate batches of surgical equipment (including viscoelastic material and IOLs) [4, 48]. Intracameral antibiotics should be used as they significantly lower the risk of endophthalmitis [47]. Furthermore, we advise that the immediate sequential approach is abandoned if complications arise during surgery on the first eye.

## Figures and Tables

**Table 1:** Characteristics of included studies.

Study ID	Methods	Participants	Interventions	Outcomes	Notes
Lundström et al. 2006 [23]	RCT	From Blekinge Hospital, Karlskrona, Sweden Inclusion: age-related cataract Group 1: *n* = 50, mean age 72.5, 54.0% women, median VA 0.6/0.6 (right/left eye) prior to surgery Group 2: *n* = 46, mean age 72.5, 54.3% women, median VA 0.6/0.6 (right/left eye) prior to surgery Excluded after randomization: 6% in Group 1 and 10.9% in Group 2 No lost to follow-up: not reported	Group 1: ISBCS Group 2: sequential bilateral cataract surgery delayed by 2 months All had phacoemulsification	VA was 0.8 or better in 91.5% of patients in Group 1 and 91.3% of patients in Group 2. Two months after surgery total disability score (Catquest score): 7.0 in Group 1 and 7.0 in Group 2.	The study was supported by the County Council of Blekinge. No conflict of interests is noted.

Sarikkola et al. 2011 [25]	RCT	From Helsinki University Eye Hospital, Helsinki, Finland Inclusion: age-related Group 1: mean (SD) age 75.3 (7.9), 73.6% women, preop CDVA (median) 20/60 Group 2: mean (SD) age 75.0 (8.1), 74.3% women, preop CDVA (median) 20/60 Excluded after randomization: 4% in Group 1 and 2.7% in Group 2 No lost to follow-up: 3.2% in total	Group 1: ISBCS Group 2: delayed sequential bilateral cataract surgery All had phacoemulsification	Postop VF-7: 24.3 (21.0) in Group 1 and 23.8 (19.2) in Group 2. Any complication (intraoperative up to 1 month postop): 106/493 in Group 1 and 124/506 in Group 2. Serious complications: 9/493 in Group 1 and 9/506 in Group 2. CDVA: 20/25 or better in 376/493 in Group 1 and 336/506 in Group 2.	The study was supported by private and public research grants. No conflict of interests was reported.

Serrano-Aguilar et al. 2012 [24]	RCT	From several clinics on the Canary Islands, Spain Inclusion: age-related cataract Group 1: mean (SD) age 72.9 (8.2), 61.2% women, preop CDVA (median) 20/100 Group 2: mean (SD) age 71.7 (7.9), 60.5% women, preop CDVA (median) 20/100 Excluded after randomization: 5.0% in Group 1 and 3.7% in Group 2 No lost to follow-up: 0 patients	Group 1: ISBCS Group 2: delayed sequential bilateral cataract surgery All had phacoemulsification	Postop VA was reported as median. Any complication (intraoperative and postop + dry eyes): 39/834 in Group 1 and 59/780 in Group 2. Serious complications: 10/834 in Group 1 versus 3/780 in Group 2. VF-14: 93.3 (12.8) in Group 1 and 95.8 (8.5) in Group 2 one month after surgery on the last eye.	The study was supported by public research grants.

CDVA: corrected distance visual acuity. ISBCS: immediate sequential bilateral cataract surgery. No: number. Postop: postoperative. SD: standard deviation. VA: visual acuity. VF-7: visual function questionnaire 7. VF-14: visual function questionnaire 14.

**Table 2:** Risk of bias in included studies.

Bias	Study ID		
	Lundström et al. 2006 [23]	Sarikkola et al. 2011 [25]	Serrano-Aguilar et al. 2012 [24]
Random sequence generation (selection bias)	Unclear risk “The patients were randomly assigned to ISCS or to DSCS.” No further description of randomization procedure	Low risk “Randomization was performed using sealed envelopes after the preoperative examination”	Low risk “A computer-generated sequence was used”

Allocation concealment (selection bias)	Unclear risk Not described in paper	Unclear risk Patients (and staff) knew after the preoperative assessment but before the surgery to which group they belonged	Unclear risk “Random numbers were obtained for all patients on the waiting list before participants were selected on the basis of the inclusion and exclusion criteria. Randomization was performed sequentially for blocks of 200 patients.” Unclear whether those including the patients in the study were aware of the randomization status before inclusion/exclusion

Blinding of participants and personnel (performance bias)	High risk Not possible to blind patients or personnel to whether the patient had ISBCS or different date bilateral surgery	High risk Not possible to blind patients or personnel to whether the patient had ISBCS or different date bilateral surgery	High risk Not possible to blind patients or personnel to whether the patient had ISBCS or different date bilateral surgery

Blinding of outcome assessment (detection bias)	Unclear risk Not reported	Unclear risk Not reported	Unclear risk Not reported

Incomplete outcome data (attrition bias)	Unclear risk High rate of exclusion after randomization/drop-outs (8/96 = 8.3%), not possible to assess whether this influenced the outcome since the characteristics of drop-outs were not compared to non-drop-outs	Low risk 96.0% in Group 1 and 97.3% in Group 2 were treated per protocol. 491/507 randomized patients had 1 month follow-up	Low risk Low number of exclusions and drop-outs (<5% at the 1 month postoperative examination)

Selective reporting (reporting bias)	Low risk Important outcomes were reported	Low risk Important outcomes were reported	Low risk Important outcomes were reported

Other bias	Low risk Not likely in this study	Low risk Not likely in this study	Low risk Not likely in this study

Risk of bias was assessed according to the Cochrane Handbook [15].

**Table 3:** Characteristics of excluded studies.

Study ID	Reason for exclusion
Akçay et al. 2013 [49]	Retrospective study evaluating the outcome after ISBCS. Not comparing to a group of patients undergoing cataract surgery on different dates.

Arshinoff et al. 2003 [50]	Retrospective study reporting the outcome after ISBCS. Not comparing to patients undergoing different-day bilateral surgery.

Arshinoff and Chen 2006 [10]	Observational study assessing the resource utilization and economic incentives of ISBCS and different date bilateral cataract surgeries.

Arshinoff and Odorcic 2009 [27]	Review summarizing published complications after ISBCS. Providing safety recommendations when operating on both eyes on the same date.

Arshinoff and Bastianelli 2011 [35]	Literature review of reported cases of postoperative endophthalmitis. Not prospective or randomized.

Arshinoff 2012 [4]	Commentary on ISBCS, the pros and cons. Not reporting the outcome after surgery in specific patients but rather in broad, general terms and referring to previously published papers on the matter.

Behndig 2009 [6]	Editorial describing the pros and cons of ISBCS.

Chung et al. 2009 [51]	Prospective, nonrandomized study comparing the outcome after ISBCS to bilateral surgery separated by 2 days. The authors found no difference in visual acuity or refractive target (96.8% and 97% were within 1 diopter of target in immediate and delayed bilateral surgery, resp.).

Covert et al. 2010 [52]	Retrospective chart review of refractive precision in patients undergoing bilateral cataract surgery. Not comparing immediate versus delayed bilateral cataract surgery.

Friström and Lundh 2005 [53]	Randomized trial comparing implantation of different IOLs on color contrast sensitivity. All patients having ISBCS. Not comparing to a group not operated on both eyes on the same date.

Henderson and Schneider 2012 [7]	Commentary discussing the pros and cons of ISBCS. Not reporting the outcomes of specific patients undergoing immediate or delayed bilateral surgery.

Huang et al. 2007 [54]	Retrospective observational study describing the outcome after ISBCS in patients undergoing surgery in general anesthesia. Not comparing to patients being operated on, on separate dates.

Jivrajka et al. 2012 [41]	Prospective study comparing refractive outcome after bilateral cataract surgery when the refractive result of the first eye was taken into consideration. Not reporting results after ISBCS.

Johansson and Lundh 2003 [55]	Retrospective study reporting the outcome after ISBCS surgery but not comparing to different date bilateral surgery.

Johansson 2004 [42]	Retrospective study reporting the refractive outcome after ISBCS but not comparing to different date bilateral surgery.

Leivo et al. 2011 [12]	Randomized trial. Comparing economic costs not the rate of complications, postoperative anisometropia, postoperative visual function, or patient satisfaction.

Li et al. 2014 [9]	Editorial computing and commenting on the risk of bilateral functional blindness after ISBCS.

Lundström et al. 2009 [11]	Observational study reporting the resource utilization in ISBCS versus different date bilateral cataract surgery.

Nassiri et al. 2009 [56]	Prospective, nonrandomized, observational study comparing the outcome after ISBCS or different date bilateral cataract surgery.

Özdek et al. 2005 [33]	Case report describing bilateral endophthalmitis after ISBCS.

Puvanachandra and Humphry 2008 [32]	Case report describing bilateral endophthalmitis after ISBCS.

Ramsay et al. 1999 [57]	Retrospective study reporting the outcome after ISBCS. Not comparing to patients being operated on, on separate dates. Only a small number of patients having phacoemulsification, the majority having ECCE.

Rosen 2012 [58]	Editorial commenting on ISBCS.

Sarikkola et al. 2004 [43]	Retrospective study reporting the outcome after ISBCS. Not comparing to a group operated on, on two separate dates.

Sharma and Worstmann 2001 [59]	Observational study reporting the outcome after ISBCS but not comparing to patients being operated on, on separate dates. Only 1 patient receiving phacoemulsification, the rest having ECCE.

Totan et al. 2000 [60]	Retrospective study reporting the outcome after ISBCS in pediatric and adult patients. Not comparing to an adult group operated on, on two separate dates.

Wertheim and Burton 2002 [61]	Observational study reporting the outcome after ISBCS. Not comparing to different-day bilateral surgery.

**Table 4:** Quality assessment and summary of findings.

Outcomes	Number of participants (studies) Follow-up	Quality of the evidence (GRADE)	Relative effect (95% CI)	Anticipated absolute effects	
				Risk with bilateral surgery on different days	Risk difference with immediate sequential bilateral cataract surgery (95% CI)
Any postoperative complications	2613 (2 studies)	⊕⊕⊝⊝ *Low* ^1,2^ Due to risk of bias and inconsistency	*RR 0.76* (0.55 to 1.07)	*142 per 1000 *	*34 fewer per 1000* (from 64 fewer to 10 more) patients were diagnosed with any postoperative complication in the ISBCS group

Serious postoperative complications	2613 (2 studies)	⊕⊝⊝⊝ *Verylow* ^1,2,3^ Due to risk of bias, inconsistency, and imprecision	*RR 1.63* (0.55 to 4.78)	*9 per 1000 *	*6 more per 1000* (from 4 fewer to 35 more) were diagnosed with a serious postoperative complication in the ISBCS group

Subjective visual function test (VF-7 or VF-14 questionnaire)	2096 (2 studies)	⊕⊕⊕⊝ *Moderate* ^1^ Due to risk of bias			The mean subjective visual function (VF-7 or VF-14 questionnaire) was *0.01 standard deviations higher* (0.47 lower to 0.48 higher) in the group randomized to ISBCS

The *corresponding risk* (and its 95% confidence interval) is based on the assumed risk in the comparison group and the *relative effect* of the intervention (and its 95% CI).

CI: confidence interval; RR: risk ratio; ISBCS: immediate sequential bilateral cataract surgery; VF: visual function.

GRADE working group grades of evidence are as follows.

*High quality*: further research is very unlikely to change our confidence in the estimate of effect.

*Moderate quality*: further research is likely to have an important impact on our confidence in the estimate of effect and may change the estimate.

*Low quality*: further research is very likely to have an important impact on our confidence in the estimate of effect and is likely to change the estimate.

*Very low quality*: we are very uncertain about the estimate.

^1^Studies were not blinded to outcome assessment.

^2^Very large differences between studies in the reported rates of complications.

^3^Studies do not have the sufficient size to reliably assess the number of serious but rare complications (e.g., endophthalmitis).
